# Automatic Representative View Selection of a 3D Cultural Relic Using Depth Variation Entropy and Depth Distribution Entropy

**DOI:** 10.3390/e23121561

**Published:** 2021-11-23

**Authors:** Sheng Zeng, Guohua Geng, Mingquan Zhou

**Affiliations:** School of Information Science & Technology, Northwest University, Xi’an 710127, China; shengzeng@stumail.nwu.edu.cn (S.Z.); ghgeng@nwu.edu.cn (G.G.)

**Keywords:** information entropy, viewpoint selection, cultural relic, Bag-of-Words

## Abstract

Automatically selecting a set of representative views of a 3D virtual cultural relic is crucial for constructing wisdom museums. There is no consensus regarding the definition of a good view in computer graphics; the same is true of multiple views. View-based methods play an important role in the field of 3D shape retrieval and classification. However, it is still difficult to select views that not only conform to subjective human preferences but also have a good feature description. In this study, we define two novel measures based on information entropy, named depth variation entropy and depth distribution entropy. These measures were used to determine the amount of information about the depth swings and different depth quantities of each view. Firstly, a canonical pose 3D cultural relic was generated using principal component analysis. A set of depth maps obtained by orthographic cameras was then captured on the dense vertices of a geodesic unit-sphere by subdividing the regular unit-octahedron. Afterwards, the two measures were calculated separately on the depth maps gained from the vertices and the results on each one-eighth sphere form a group. The views with maximum entropy of depth variation and depth distribution were selected, and further scattered viewpoints were selected. Finally, the threshold word histogram derived from the vector quantization of salient local descriptors on the selected depth maps represented the 3D cultural relic. The viewpoints obtained by the proposed method coincided with an arbitrary pose of the 3D model. The latter eliminated the steps of manually adjusting the model’s pose and provided acceptable display views for people. In addition, it was verified on several datasets that the proposed method, which uses the Bag-of-Words mechanism and a deep convolution neural network, also has good performance regarding retrieval and classification when dealing with only four views.

## 1. Introduction

High-precision 3D cultural relics allow researchers and viewers to observe surface morphology and local features from an arbitrary angle. People are often more interested in a few views after appreciating cultural relics from different perspectives. The viewing experience for tourists could be improved by providing several representative views for each cultural relic. These views are also suitable for labeling cultural relics, in order to include richer semantic information in the labels. The automatic selection of representative views of cultural relics is a crucial stage in building a wisdom museum. Selecting the set of best views is an NP-hard problem [1] and complex surfaces will also have an important impact on the method. Papadimitriou explained “spatial complexity” and effectively examined the various behaviours of complex systems [2]. The Euler characteristic is calculated to measure the spatial complexity of the 3D spatial object. There is still a degree of difficulty in describing most cultural relics with a higher genus. The goal is to display each cultural relic with the minimum possible number of views. It is also important to estimate whether the selected set of views contains peculiar features. Accurately identifying a cultural relic relies on marking more detailed information from the different views, which can be used to expand the cultural relic’s knowledge base [3]. This mean that we must be able to automatically obtain several views basically covering the surface of the 3D object and include significant features on the high-precision 3D model of the cultural relic.

Bonaventura et al. reviewed 22 view selection methods [4], which are classified according to the area, silhouette, depth, stability and surface curvature. They then used Dutagaci’s method for evaluation [5]. Generally, oblique views between the frontal view and the profile view are often preferred as representative views for 3D objects. However, one view is not enough for understanding the whole 3D model, while some methods also provide multi-view selection schemes [6,7,8]. It is still difficult to obtain a few views with large shape differences, such as the views containing the front as well as the side of the object.

**Figure 1:**
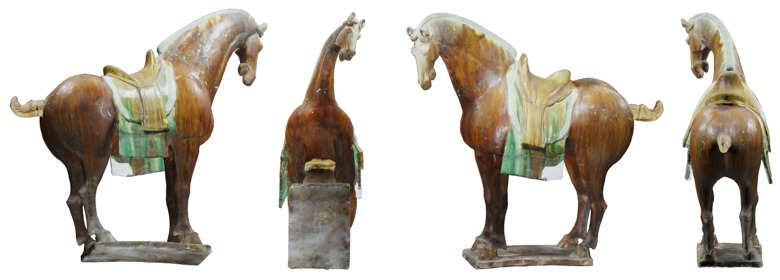
Representative view of the horse.

To solve these problems, two new measures based on information entropy have been defined. The first one is referred to as depth variation entropy. The larger the entropy, the closer to seeing more depth swings. The second one is referred to as depth distribution entropy. The greater the entropy, the closer to seeing more depth quantity, which is a similar result to that of the depth distribution [9]. Based on this, we designed a framework for multi-view selection, in which continuous views are captured by placing cameras on the vertices of a geodesic unit-sphere to calculate the depth variation entropy and depth distribution entropy for choosing the depth map with the maximum entropy. The multi-view selection method is then proposed for automatically achieving four viewpoints far away from each other. The method can ensure that the views obtained by a 3D model in an arbitrary pose are consistent, which highly reduces the workload of manual model alignment. Moreover, the obtained views show the cultural relics in a near panoramic view, and therefore, we can see not only the side with a large projection area but also the front side with a small projection area. As shown in Figure 1, the obtained views can present not only the side of the horse, but also its front.

In addition, a quantitative method has been proposed to analyze a set of views. Among a large number of view-based 3D model retrieval and classification algorithms, it has been deduced that multi-view algorithms can distinguish different types of models. The large number of views used in these methods cannot meet the needs of view selection for the cultural relic model. However, the quantitative evaluation method is also suitable for analyzing and evaluating the representation capacity of the 3D model views. The word bag mechanism was adopted to build a codebook in the selected views and a threshold word histogram was proposed to analyze whether the selection views have enough features to represent the whole model by carrying out 3D model retrieval. Our four selection views for retrieval also had good performance with public datasets.

In the experiment, we present the best views selected by four different algorithms and show the results obtained by the proposed multi-view selection method. Afterwards, our threshold word histogram was used to represent a 3D model, and the 3D model retrieval was carried out on the McGill Shape Benchmark (MSB) [10] and the Princeton Shape Benchmark (PSB) [11] to analyze the feature representation capacity of the different views. Finally, the multi-view classification method compared the recognition effects of different numbers of views on the ModelNet40 dataset [12]. Our four views demonstrated the general applicability of the proposed approach, in line with people’s habits of looking at items from all around.

The rest of this research is organized as follows. In Section 2, we illustrate the related work. In Section 3, we propose our method and analyze its superiority. In Section 4, the experimental results on a comparison of view selection methods, 3D shape retrieval and recognition are described. Finally, we present our conclusion in Section 5.

## 2. Related Work

In this section, we review related work in two different categories related to the techniques presented in this paper. In the first part, we examine studies related to viewpoint selection of a 3D object. Afterwards, we review related work in the area of object classification and retrieval.

### 2.1. Selection of the Best View of 3D Objects

Plemenos and Benayada [13] studied the projected area of a model from a viewpoint as a measure of viewpoint goodness. Vázquez et al. proposed viewpoint entropy to define the goodness of a view [14]. The view with maximum entropy had the maximum visual information, while most of the depth information was lost. Stoev and Strasser introduced an approach for computing an optimal camera position to visualize terrain [15]. They used not only the projected area but also the depth of the image to find a good view.

Page et al. proposed curvature entropy and silhouette entropy to measure the goodness of a view [16]. Lee et al. suggested using mesh saliency based on the local curvature over the surface to investigate the best view [17]. Polonsky et al. analyzed a number of the best view selection algorithms and concluded that a combination of descriptors would amplify the respective advantages of different view descriptors [18].

Vázquez computed the stability of a viewpoint by comparing the viewpoint with its neighbors [19]. Vieira et al. proposed a learning approach for imitating the user by pre-selecting good views [20]. Secord et al. leveraged the results of a large user study to obtain people’s preferred views [9]. Bonaventura et al. defined three types of viewpoint information to quantify the information associated with each viewpoint [21]. Dugataci et al. used the vertices of the geodesic sphere to sample 258 viewpoints on the viewing sphere of a model [5]. The difference was measured by the geodesic distance between the optimal viewpoint and the ground truth of 26 participants. They provided a way to evaluate whether the best view was closer to human preferences. Bonaventura et al. elaborated on a review of 22 measures to select good views of a polygonal 3D model [4]. For more information about the development of viewpoint selection methods, we refer the reader to this survey.

### 2.2. View-Based 3D Model Retrieval and Classification

To develop a method to quantitatively evaluate the representativeness of multiple views, we drew lessons from the technologies related to view-based 3D model retrieval and classification. The multi-view algorithm explores the object’s information from different visual perspectives, puts the 3D model in a sphere and observes it in the center of the sphere from different viewpoint angles. This way of analyzing the characteristics of a 3D model imitates the behavior of human object recognition [22]. There are many view-based 3D model retrieval and classification methods, but we mainly introduce the methods related to this paper.

The light field descriptor (LFD) method places the cameras on 20 vertices of a 12-hedron. Since the silhouette projected from opposite vertices is the same, 10 images are represented for 20 viewpoints. They finally calculate the Zernike moment and Fourier coefficient of the 100 images to form descriptors for a 3D model [23]. Shih et al. proposed elevation descriptors that are robust to rotation, which are extracted from the elevation information of six different views (front, top, right, rear, bottom and left) [24]. Chaouch et al. first solved the normalization problem of 3D objects, and then represented the 3D model by 20 depth images [25]. Ohbuchi et al. extracted the salient local features (SIFT) from each range image and integrated them into a histogram using the Bag-Of-Features (BoF) approach. They compared the performance of different view selections and finally recommended 42 views for extracting features [26]. Daras and Axenopoulos created a compact representation of a 3D object as a set of multiple 2D views including silhouette and depth images. Their experiments showed that the 18-view representation of a 32-hedron produces better retrieval results than the 6-view representation of the 8-hedron [27]. Lian et al. described each view as a word histogram and the objects were compared by clock matching (CM-BOF). The number of views was chosen as 66 after they investigated the influence of the number of views on retrieval performance [28].

With the rise of deep learning, the methods of recognizing 3D model from multi angle views have also developed greatly, and the most representative work is multi-view convolutional neural networks (MVCNN) [29]. The authors raised a camera 30 degrees from the ground and obtained 12 views at intervals of 30 degrees. In the conclusion, it was proposed that different combinations of 2D views still needed to be explored. In addition, there are many depth learning methods, most of which focus on the improvement of network structure and the choice of views [30,31]. Basically, the viewpoints on the vertex of a regular polyhedron are adopted. Similarly, more views have better results.

For 3D cultural heritage classification, Hristov et al. developed a software system for classifying archaeological artefacts represented by 2D archaeological drawings [32]. Gao et al. transformed the scale-invariant heat kernel signature descriptor into a low-dimensional feature tensor by the Bag-of-Words mechanism to solve the problem of samples in the dataset that lack category labels [33]. Many other classification methods have also tried to find more representative features to achieve a more precise result [34,35,36]. High precision and few samples are the characteristics of cultural relics’ data, and these are also the key factors to be considered in practical applications.

We can see that many kinds of application based on views have two main strategies: optimal view selection and uniform selection. A few views can represent a model in selection methods aiming to achieve the best views. A large number of views are needed to achieve good retrieval and classification results in general. For a high-precision and scarce 3D model of artistic relic data, we propose a multiple optimal view selection method and a quantitative evaluation method for multi-view comparison.

## 3. The Proposed Method

### 3.1. Depth Variation Entropy and Depth Distribution Entropy

There are two obvious differences between the best views. One is the view of the object with a large projection area (e.g., the side of the horse) and the other is the view with a large depth distribution (e.g., the front of the horse) [4]. These views are often what we want to display. The capture of depth maps is efficient, even for high-resolution 3D models, and it also has rich information. We define depth variation entropy and depth distribution entropy on the depth map to obtain two similar kinds of views.

**Figure 2:**
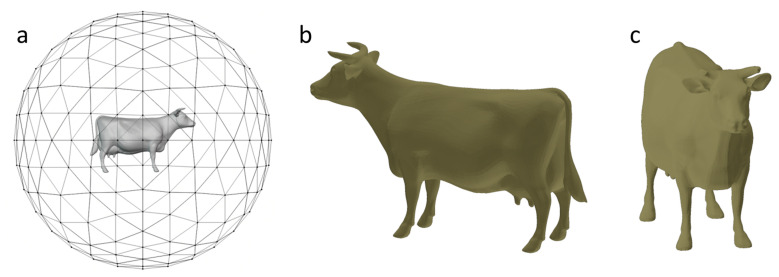
In the left (**a**), 258 cameras are placed on the vertices of the geodesic unit sphere to obtain the depth map for the cow model. The middle (**b**) and right (**c**) are the views with maximum depth variation entropy and depth distribution entropy, respectively.

Similar to [5], we sampled close to continuous 258 views, which were captured by placing cameras on the vertices of a geodesic unit-sphere generated from a regular octahedron. The cameras pointed towards the center of the bounding box of the mesh and the orthographic depth maps were rendered with a size of 223 × 223. The mesh was uniformly scaled according to the length of the diagonal of the bounding box to ensure that the depth maps fit into the viewing volume (see Figure 2a). The depth map obtained in this way is equivalent to the picture viewed from the equidistant positions around the model; it is also closer to the human habit of observing objects.

Discrete Shannon entropy can be used to measure information, and asymmetric entropy can be used to reveal a more profound evolution of ecological communities and populations [37]. Let $X=\{x_{1},x_{2},\ldots ,x_{n}\}$ be discrete random variable and $p_{i}$ the probability of $x_{i},i=1,\ldots ,n$. The Shannon entropy of the discrete random variable *X* is defined as $H\left(X\right)=-\sum_{i=1}^{n}p_{i}logp_{i}$. Based on the Shannon entropy, Vázquez et al. proposed the definition of viewpoint entropy. They used a probability distribution of the relative area of the projected faces over the sphere of directions centered on the viewpoint [14]. We define two similar measures based on depth information. Each pixel of the depth map stores the orthogonal distance of a viewpoint to the surface. The first measure uses the neighborhood information of a central pixel using a window of fixed size (blank pixels are not included). The dispersion *D* of each pixel in the depth map is the ratio of the variance to the mean in a 3 × 3 window. Given a depth map *V*, the depth variation entropy is:

(1) $$H\left(V\right)=-\sum_{i=1}^{n}\frac{D_{i}}{D_{t}}log\frac{D_{i}}{D_{t}}$$

where *n* is the number of the visible depth pixels of the depth map and $\sum_{i=1}^{n}\frac{D_{i}}{D_{t}}=1$, $D_{i}$ is the dispersion of pixel *i* and $D_{t}$ is the total dispersion value of the depth map. Given $a=\{a_{1},\ldots ,a_{m}\}$ be a set of a pixel value and its neighborhood, *m* is the number of the visible depth pixels in the 3 × 3 window. The dispersion D of each pixel is $D_{i}=v\left(a\right)/<a>$, where v(·) is the variance and <·> is the mean value. This formula uses the ratio of each pixel depth dispersion to the total as the probability distribution to compute the entropy. This measure is sensitive to noise, so mean filtering must be carried out first when calculating the entropy. It should be noted that the pixels on the visible depth boundary should not be affected by the blank pixel value during filtering.

The second measure uses the depth distribution of the depth map. The depth distribution is defined as $1-\int S{\left(z\right)}^{2}dz$ in [9], where *z* is the depth and *S* is the normalized histogram of the depth. It encourages objects with largely planar areas to take oblique rather than head-on views, and is insensitive to noise. We calculate the depth distribution on discrete viewpoints and define it in connection with Shannon entropy. Given a depth map *V*, The visible depth value stroed in each pixel are classified into *n* bins to form a depth histogram on a depth map. let each bin value of the normalized depth histogram as a probability, the occurrence number of each bin is $F=\{F_{1},\ldots ,F_{n}\}$, the depth distribution entropy is:

(2) $$H\left(V\right)=-\sum_{i=1}^{n}\frac{F_{i}}{F_{t}}log\frac{F_{i}}{F_{t}}$$

where *n* is the number of visible depth bins of the depth map and $F_{t}$ is the total occurrence number of the depth value and $\sum_{i=1}^{n}\frac{F_{i}}{F_{t}}=1$. Our maximum depth distribution entropy result is similar to the result of [9] but, theoretically, the maximum entropy of our depth map has the maximum depth fluctuation information.

The maximum depth variation entropy or depth distribution entropy is obtained when a certain view has the same probability distribution. Because our views were captured on a unit-sphere, which limits the position of the collected views, they may not have reached maximum entropy. However, in the obtained views, it was found that with greater depth variation entropy, more depth swings can be seen, and with greater variance entropy, more different depths can be seen. Figure 2b,c shows the results obtained with this method for the cow model. Figure 2b shows the side view of the cow and Figure 2c shows the front view of the cow; these correspond to the classical “three-quarter view” of an object. These two views are exactly what we want to show to museum visitors.

The views obtained by our two metrics should have less correlation. Xiaojun Zhao et al. propose an effective method to detect nonlinear correlation [38]. Due to each depth histogram represent the information of this view to some extent. We regard the set $F=\{\frac{F_{1}}{F_{t}},\ldots ,\frac{F_{m}}{F_{t}}\}$ as a 1-dimensional time series on a depth map to compute mutual information. Different from the number *n* in Formula (1), *m* is the length of the minimum depth to the maximum depth in all views of a model. We can obtain a 258-dimensional time series *G* of equal length *m* on a 3D mesh. The incorrectness of two time series can be quantified by the Kullback-Leiber divergence. We use the method defined by them to obtain the mutual information matrix of the mesh in Figure 2. The overall mutual information mean value of 258 depth maps is 0.4587 and the mutual information between Figure 2b,c is 0.1565. While the mutual information between the views obtained by using our two measures is not the most irrelevant, the correlation is obviously low, verifying that the views with large differences can be obtained by our two measures from a quantitative point of view.

### 3.2. Multi-View Selection

**Figure 3:**
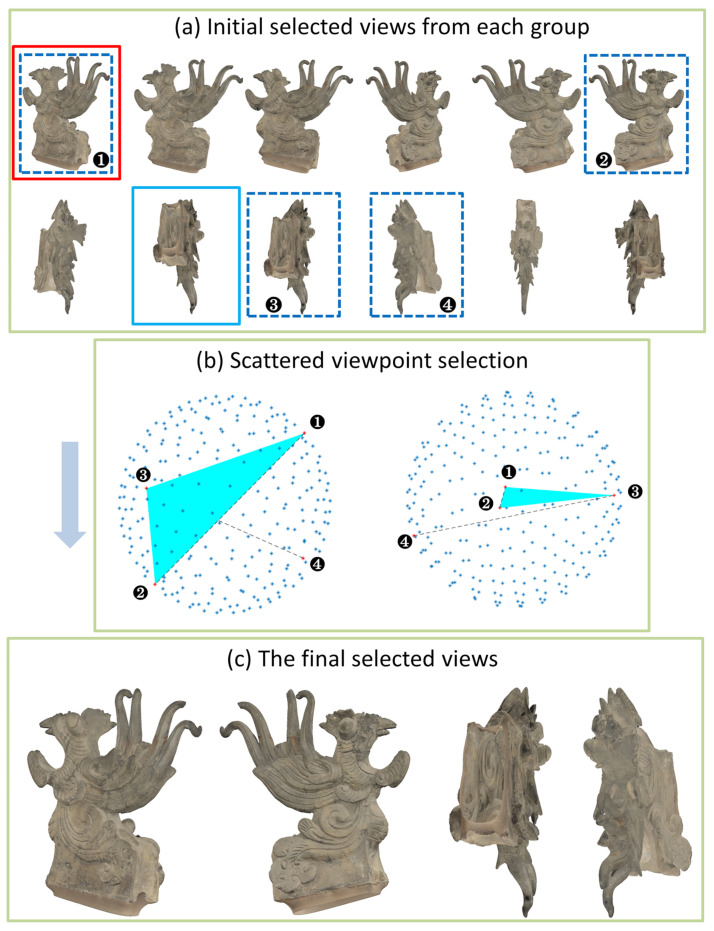
The whole pipeline of Representative View Selection of Jishou. Initial selected views from each group are shown in (**a**). The viewpoints in the order of marks 1, 2, 3 and 4 in (**b**) were obtained according to the scattered viewpoint selection rules. The final four selected views are shown in (**c**).

While the measure we defined seems to provide a good view for visitors, it cannot always guarantee excellent results with different meshes. In the example of the strangely shaped relic named Jishou shown in the Figure 3a, the views marked with the solid red wireframe are the results of maximum depth variation entropy, and the solid blue wireframe shows the results of maximum depth distribution entropy. Because one can see the inside through the open base, the solid blue wireframe has the maximum depth entropy. This shows that it is difficult to provide a good display for all models with only two views. In addition, the depth maps obtained with different poses are different, which leads to diversity in the results. In order to improve the overall stability, we propose a method with scattered viewpoints for selecting the best views. This method ensures that the results obtained with arbitrary poses are consistent, which eliminates the step of manually adjusting the attitude of a mesh. At the same time, the four views obtained can basically display most of the visual information of the 3D object.

We first normalized the objects by principal component analysis (PCA) [39]. Since pose alignment processing by PCA cannot ensure that the object has the same orientation as the principal axes, Daras and Axenopoulos chose the rotated object with the minimum bounded volume after both PCA and visual contact area (VCA) analysis [27]. Moreover, Lian et al. combined PCA and the rectilinearity to obtain better normalization results [28]. However, we only need the models with arbitrary poses to be orthogonal to each other after being converted. As long as the collected viewpoint can be rotated by 90 degrees and remain unchanged, the captured depth map is still consistent. While there will be rotation in the obtained depth map, it will have no effect because the method is rotation-invariant in this study.

After pose normalization, we divided the unit-sphere into three planes formed by the *x*–*y*–*z* coordinate axes into eight parts to make a group with 45 viewpoints. There are duplicate viewpoints on the great circle between each adjacent group. This grouping ensures that the viewpoints belonging to each part are consistent after 90-degree rotation of the object. It is possible that the best view of each group will repeat. In extreme cases, the best view is in the middle of the hemisphere, which contains four groups. That also means that the number of best views with our two measures is 4–16.

We reduced the number of best views further. Four scattered viewpoints that are far away from each other were selected in order to obtain a macro-view of the whole. Given the best view sets v1 of depth variation entropy and v2 of depth distribution entropy, the following basic steps of the procedure are performed:

Step 1. Remove the duplicate views in the acquired view; there will be at least four views after this step.
Step 2. Calculate the direction vector from the spherical center point to each viewpoint position and find the two viewpoint positions belonging to v1 with the largest included angle between their direction vectors.
Step 3. In v2, calculate the triangle area composed of each viewpoint position and the two determined viewpoints’ positions. The vertex that maximizes the area of the triangle is the third choice.
Step 4. Find the fourth viewpoint position in v2 with the largest angle for the third selected viewpoint position.

Figure 3a shows the 12 best views of Jishou. The blue dotted box indicates the selected view, in which the duplicate views have been removed. Figure 3b is a sketch map for assisting in understanding the selection rules of decentralized views. Marks 1, 2, 3 and 4 in the figure represent the order of the selection rules. The third view was selected by the maximum triangle area, which inspired by the definition of a solid angle [40]. The solid angle is computed by projecting an object onto the unit-sphere and measuring the area of its projection. The area of the triangle is obviously proportional to the area projected onto the sphere. The more viewpoints tend to be scattered, the larger the area of the triangle. The area of the regular triangle on the great circle of the sphere is the largest, and the distribution of the three vertices is also the most dispersed. Figure 3c shows the four views in the final selection. While the shape of Jishou is peculiar, we can still obtain a comprehensive understanding of the model without bad results.

### 3.3. Threshold Word Histogram Method for Representative Analysis

In addition to find the view conforming to human aesthetics, it is also important to quantitatively analyze whether the multi-view features are enough to represent the 3D model. In view-based 3D model recognition and retrieval tasks, researchers have focused on how to improve the feature extraction capacity and obtain as many of the peculiar features of a model as possible to achieve good results. Our purpose was to analyze whether a small number of representative views could represent the model. Based on previous methods, we propose a threshold word histogram construction method for analyzing the representative capacity of different multi-views.

The deep learning method has strong feature extraction capacity and can directly input different views in the same neural network to compare the classification results (see the experiment in Section 4.2). However, we generally need a large number of samples to provide a priori knowledge. In the small samples of 3D cultural relics, the view-based feature extraction method is more suitable for analyzing and comparing different views. Ohbachi et al. extracted SIFT [41] features from multiple views and took the center of a K-means cluster as a visual codebook for quantizing the local features into a visual word [26]. Lian et al. regularize the model first and then proposed a clock matching mechanism to ensure that it compared with corresponding view, so as to improve the accuracy [28]. Since the calculating time of the K-means clustering was significant, they randomly sampled local feature vectors to create the codebook in their method. Both of these studies verified that the more views, the better the retrieval accuracy, but, considering the amount of calculation, 42 and 66 views were recommended, respectively, in [26,28]. To analyze the representative capacity of a few views, we propose the threshold word histogram method. Unlike previous methods, the codebook *C* is generated from the selected views. The threshold method removes the influence of the features which are not close to *C*.

**Figure 4:**
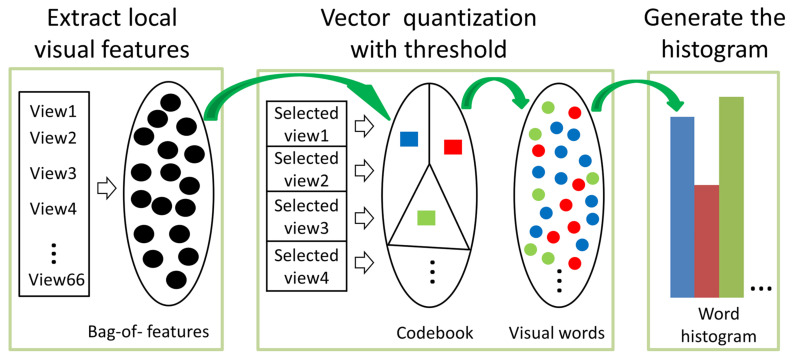
Generation of the threshold word histogram.

The biggest difficulty of model retrieval is that the best view of the classification model is not necessarily the view from the same perspective. To solve this problem, the features extracted from the selected view were used as a codebook, and then the visual vocabulary was created by setting the threshold to find the features similar to the codebook from the uniform view. The SIFT descriptor is calculated, using the VLFeat matlab source code developed by Vedaldi and Fulkerson [42]. As shown in Figure 4, we extracted the local features from 66 uniform depth maps by using the SIFT algorithm. A codebook with $N_{w}$ visual words was generated via sampling from the selected views but not clustering. Each model contributed only eight feature vectors from the target database to form a codebook, then each feature was assigned to a visual word with a threshold. The threshold was set as the mean value of the Euclidean distance between the top, front and left views and their adjacent views for each model of the target dataset. After the visual words were created, the frequencies of visual words were accumulated into a histogram with $N_{w}$ bins. Each histogram shows the $N_{w}$-dimensional feature vectors for the 3D model.

The distance between a pair of feature vectors was computed by using the histogram intersection distance presented in [28]. Let us assume that model *k* is described by the word histogram $W_{k}=\left\{W_{k}\left(j\right)|j=1,2,\ldots ,N_{w}\right\}$, then, given two word histograms $W_{1}$ and $W_{2}$, the maximum dissimilarity histogram intersection distance Dmaxhis is defined as follows:

(3) $$D_{maxhis}=1-\frac{\sum_{j=1}^{N_{w}}min\left(W_{1}\left(j\right),W_{2}\left(j\right)\right)}{max(\sum_{j=1}^{N_{w}}W_{1}\left(j\right),\sum_{j=1}^{N_{w}}W_{2}\left(j\right))}$$

The distance Dmaxhis measures the similarity between different models. The threshold word histogram provides a method for analyzing the representativeness of selected views by evaluating retrieval quality in public datasets.

## 4. Experiment Results and Analysis

To fairly evaluate our approach, we implemented the method described above and tested it on several public datasets. The experiments described in Section 4.1, Section 4.2.1 and Section 4.2.2 were performed on a desktop PC with a 2.30 GHz Intel Core i5-8300 and 8 GB of RAM. The experiments in Section 4.2.3 were performed on a desktop PC with a 6 × 2.50 GHz Intel Xeon E5-2678 and NVIDIA GeForce RTX 2080 Ti. We implemented the complete algorithm in Matlab 2018. We verified the advantages of our approach for view selection with three different 3D models. The 3D shape retrieval results were analyzed via our method, which used grouping word histograms, on the McGill Shape Benchmark (MSB), which consists of 255 objects classified into 10 categories [10] and the test set of the Princeton Shape Benchmark (PSB) containing 907 models are classified into 92 categories [11]. Finally, we compared the classification accuracy for selecting different views in the ModelNet40 database [12] versus MVCNN [29].

### 4.1. Generally Applicability of Multi-View Selection

In the first experiment, we present the best views selected from the 258 views described in Section 3.1 by four different algorithms for the Hu relic (256,000 triangles) and the Goat relic (800,000 triangles), then show the results of our multi-view selection method.

**Figure 5:**
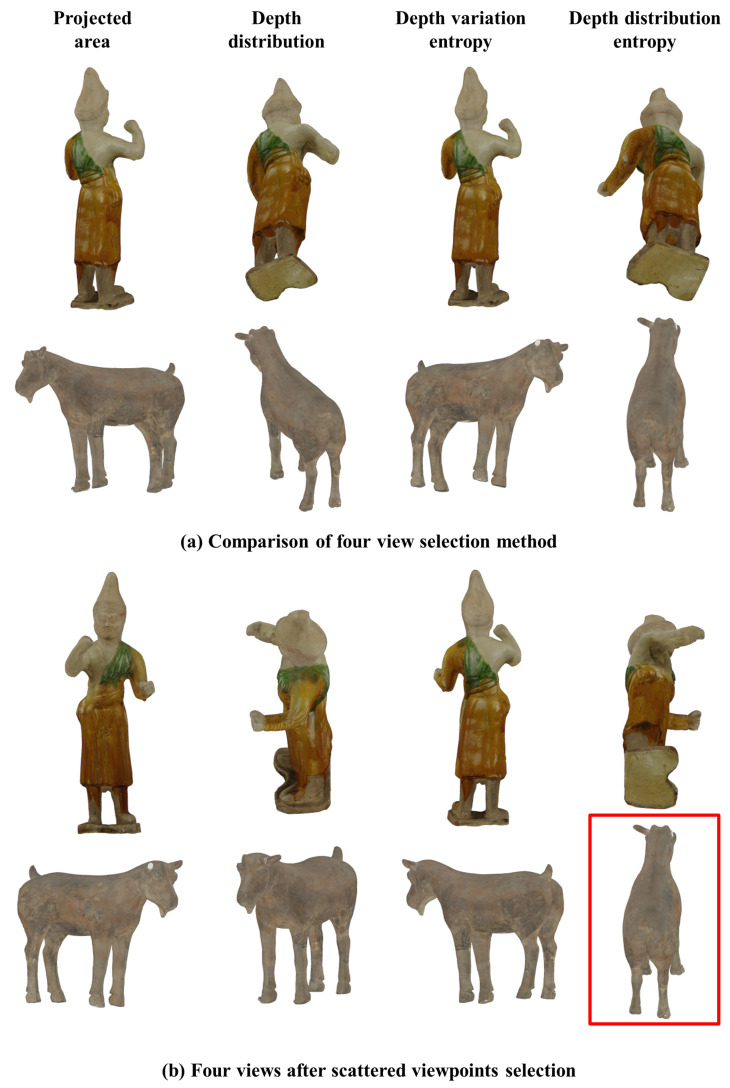
Comparison of optimal view methods and scattered view selection results.

As shown in Figure 5a, in the views obtained by the projected area method, one can see a larger area in the first column [13], which conforms to the preferences of visitors regarding the goat but shows the back of the Hu. The depth distribution method [9] in the second column produces a four-quarters view of the goat, but the results for the Hu are not popular because people prefer to see the front of an object.

Our depth variation entropy method in the third column provides a similar view to the projected area method for the Hu and the goat. Slightly oblique views were obtained but the backs of the objects were still shown. Our depth distribution entropy method in the fourth column was similar to the depth distribution method, which produced a slightly worse view for the Hu because the inclination of the base had more specific gravity. Note that it is difficult to achieve good results for all models with one method.

Figure 5b shows the results of our multi-view selection algorithm. We used four scattered views to represent a 3D model. Near ideal results were obtained for the two models, which show not only the side but also the front. While there is not one ideal view of the Hu from bottom to top, observations from this view also complement the cognition of the whole object. The views marked by the red box in the figure are the results of maximum depth distribution entropy. No matter whether the maximum entropy view is selected or not, we achieve the views that are similar to looking around an object. This verifies the effectiveness of the method for producing good results, demonstrating the generally applicability of our approach.

### 4.2. Evaluation of Small Number Views to Represent a 3D Model

In this experiment, we first compared our method with several other unsupervised 3D model retrieval algorithms and then used the selection of different views to analyze the 3D model representation ability of different views.

#### 4.2.1. Evaluation of the Threshold Word Histogram Method

Nearest neighbor (1-NN), first-tier (1-Tier), second-tier (2-Tier) and discounted cumulative gain (DCG) were used to compare our method with the approaches of CM-BOF [28], LFD [23], radialized spherical extent function(REXT) [43], spherical harmonic descriptor(SHD) [43], gaussian euclidean distance transform(GEDT) [44], viewpoint information I2 [21] and D2 shape distribution (D2) [45] on the PSB test set with base classification. The benchmark data for comparison come from [11,28]. As shown in Table 1, the CM-BOF of 66 views was the best at all levels. The data of our word histogram in for views was the closest to the LFD of thousands of contours, which was better than the results for REXT, SHD, GEDT, I2 and D2. It can be seen that the effect of the algorithm was basically the same on the two datasets. The algorithm that obtained better results needed more views, and we only used four views to obtain close results to the LFD. The values of 1-NN, 1-Tier and 2-Tier were very close, but the DCG decreased. This indicates that the first few results were returned accurately during retrieval with fewer views, but there was more confusion when all are recalled. In the same class, data with large shape changes were difficult to classify correctly when there were few views. In order to analyze the applicability of a small number of views, we made further experiments.

#### 4.2.2. Applicability of a Small Number of Views

**Figure 6:**
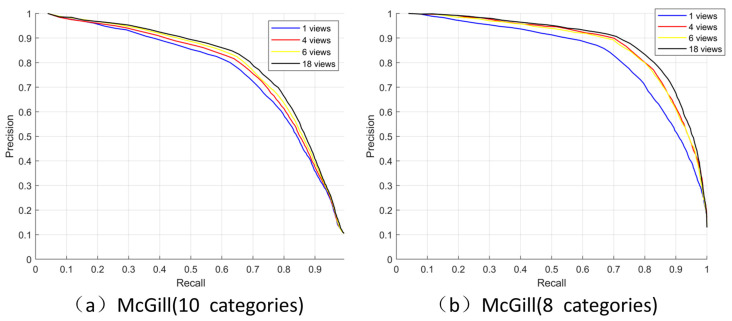
Precision-recall curves of four different views.

We use different numbers of views (1, 4, 6 and 18) and used the word histogram to analyze the McGill dataset. The selection of one view was obtained by using the maximum projected area, and four views were selected by our method. The 6 views were front, back, up, down, left and right, and the 18 views were obtained by placing the camera at the 18 vertices of the 32-hedron subdivided once from the regular octahedron. The PR graph is used to intuitively analyze the retrieval with different view choices. Figure 6a shows the PR graph on the M dataset which has 255 models with 10 classes; the horizontal axis is the recall rate and the vertical axis is the accuracy. The recall rate is the correct proportion of the retrieved model, and accuracy is the correct proportion of the retrieved model as the recall rate increases. The higher the numerical value that can be maintained, the better the partition. It can be seen that the more views, the better the effect.

We found that the two classes with the worst effects on this dataset were the snakes and the octopuses. To further analyze under what kind of data can be better expressed using our four views, we removed these two categories and carried out further experiments. Figure 6b shows the test results for 203 models with eight classes. In this case, the PR diagrams for 4 and 6 views were very close. It is clear that having four views can achieve the same effect as 6 views to distinguish various models in this eight-class dataset. Table 2 illustrates this more clearly. Our four selected views exceed 6 views on 1-Tier, 2-Tier and DCG. We suggest that the reason for the confusion in the snakes class was the depth changes were relatively uniform lead to the SIFT characteristics showed relatively few uniqueness. The second reason is that the shapes of the different categories of models are very similar and it was still difficult to distinguish them with fewer views (like octopuses and spiders). The experiments showed that having more views must provide more information, but using our four views had also better representation ability when a distinct model category shape itself is obvious.

#### 4.2.3. Classification Using a Small Number of Views Based on Deep Learning

The deep learning method for model classification has weak interpretability but strong ability to extract features. To analyze the representation ability of the four views selected in this paper, different views were used for comparing the classification accuracy on ModelNet40 dataset. MVCNN recommends using 12 views to represent a 3D model, which also indicates that the selection of views remains to be explored. In order to test the recognition ability of a small number of views, we used our four views, eight views extracted from eight uniform viewpoints with an angle of 30 degrees and an interval of 45 degrees on the geodesic sphere and twelve views extracted from eight uniform viewpoints with an angle of 30 degrees and an interval of 30 degrees on the geodesic sphere. Vgg11 was selected in MVCNN, and other the super-parameters were consistent. The test results are shown in Table 3. It can be seen that the conclusion was similar to that in Experiment 2: the more views, the better the effect, and the classification accuracy of four views was close to that of more 8 views. While it is difficult for a small number of views to surpass the results of larger number of views, the close classification accuracy also shows that our selection of four views also has sufficient features. The characteristics of the cultural relic model are more distinctive, and our four views are enough to express it from the perspective of the features it contains.

## 5. Conclusions

In this study, we propose an efficient method for selecting representative views of 3D models of cultural relics, and propose a method for 3D model retrieval with a small number of views to analyze the representative capability of 3D models. Our study makes the following three main contributions: The first is that it proposes two new measures for selecting the view with the most abundant information on the depth map according to according to the characteristics of information entropy. One measure is to obtain the depth map with the most visual information, and the other is to obtain more depth distribution information. The second can be used to build a block dense viewpoint extraction model and propose a scattered viewpoint selection algorithm. This algorithm can improve the universality of view selection, make up for the problem that the optimal view may not obtain the view of interest, and is more in line with the habit of human observations of 3D objects. Third, a threshold word histogram method for 3D model retrieval with a small number of views is proposed. It reasonably analyzed the representative ability of a small number of views of 3D models, and verified the analysis results of the representative ability of a small number of views via the deep learning method.

The proposed method could efficiently produce a small number of optimal views of high-precision 3D models of cultural relics, which were not affected by the model pose and eliminated the steps of manually aligning the 3D model. The threshold word histogram method can be used to analyze the representative ability of a small number of views of the 3D model. We believe the approach described in this study will be noteworthy for researchers who are attempting to select and analyze representative views of a 3D model.

## Figures and Tables

**Table 1:** Comparing 7 shape descriptors on the PSB test set with base classification.

	CM-BOF	LFD	Ours	REXT	SHD	GEDT	I2	D2
1-NN (%)	73.1	65.7	65.3	60.2	55.6	60.3	39.4	31.1
1-Tier (%)	47.0	38.0	36.0	32.7	30.9	31.3	20.8	15.8
2-Tier (%)	59.8	48.7	46.9	43.2	41.1	40.7	27.9	23.5
DCG (%)	72.0	64.3	56.0	60.1	58.4	23.7	45.3	43.4

**Table 2:** Comparison of different data sets under four view selections.

	McGill (10 Class)				McGill (8 Class)			
Number of views	1	4	6	18	1	4	6	18
1-Tier (%)	68.9	70.7	71.8	73.0	74.1	78.6	78.6	80.5
2-Tier (%)	83.2	83.8	84.2	84.7	88.7	91.5	91.4	92.3
DCG (%)	83.4	83.9	84.3	84.7	85.8	87.1	87.0	87.5

**Table 3:** Comparison of classification accuracy on different views using MVCNN.

Number of Views	4	8	12
Classification (Overall accuracy)	85.1%	86.3%	92.5%
Classification (Mean accuracy)	81.7%	83.1%	88.9%

## Data Availability

Publicly available datasets were analyzed in this study. This data can be found here: https://shape.cs.princeton.edu/benchmark/ (accessed on 28 October 2021) and http://www.cim.mcgill.ca/~shape/benchMark/ (accessed on 28 October 2021).
